# Effect of Vitamin D Supplement on Vulvovaginal Atrophy of the Menopause

**DOI:** 10.3390/nu12092876

**Published:** 2020-09-21

**Authors:** Thawinee Kamronrithisorn, Jittima Manonai, Sakda Arj-Ong Vallibhakara, Areepan Sophonsritsuk, Orawin Vallibhakara

**Affiliations:** 1 Department of Obstetrics and Gynaecology, Faculty of Medicine, Ramathibodi Hospital, Mahidol University, Bangkok 10400, Thailand; giftgizz83@gmail.com; 2 Female Pelvic Medicine and Reconstructive Surgery Unit, Department of Obstetrics & Gynaecology, Faculty of Medicine, Ramathibodi Hospital, Mahidol University, Bangkok 10400, Thailand; jittimabartlett@gmail.com; 3 ASEAN Institute for Health Development, Mahidol University, Nakhon Pathom 73170, Thailand; dr.sakda@gmail.com; 4 Department of Clinical Epidemiology and Biostatics, Faculty of Medicine, Ramathibodi Hospital, Mahidol University, Bangkok 10400, Thailand; 5 Reproductive Endocrinology and Infertility Unit, Department of Obstetrics and Gynaecology, Faculty of Medicine, Ramathibodi Hospital, Mahidol University, Bangkok 10400, Thailand; areepan.sop@mahidol.ac.th

**Keywords:** vulvovaginal atrophy, VVA, vaginal maturation index, VMI, vaginal health, vitamin D, ergocalciferol, vitamin D supplement

## Abstract

The effects of oral vitamin D supplements on vaginal health in postmenopausal women with vulvovaginal atrophy (VVA) was evaluated. A double-blinded, randomized placebo-controlled trial was conducted for 12 weeks to investigate changes on vaginal maturation index (VMI), vaginal pH, and the visual analog scale (VAS) of VVA symptoms. The vitamin D group received oral ergocalciferol, at 40,000 IU per week, while the placebo group received an identical placebo capsule. Eighty postmenopausal women were enrolled. There were no significant differences in baseline characteristics between both groups. In an intention-to-treat analysis, VMI, vaginal pH, and VAS of VVA symptoms showed no significant differences between both groups at the six and 12 weeks. However, the mean difference of VMI in the vitamin D group between baseline and at six weeks showed significant improvement (5.5 + 16.27, *p* <0.05). Moreover, the mean vaginal pH and VAS of VVA patients in the vitamin D group were significantly improved at both six and 12 weeks compared to baseline. The oral vitamin D supplementation for 12 weeks potentially improves vaginal health outcomes in postmenopausal women with VVA symptoms, demonstrated by the improved mean VMI, vaginal pH, and VAS at six and 12 weeks between baseline, however, no significant differences were observed from the placebo treatment.

## 1. Introduction

Vulvovaginal atrophy (VVA) is common, however, many postmenopausal women are not aware of this problem. This condition results from the changes in the female reproductive system during menopause, after cesstion of ovulation and the accompanying reduction in estrogen levels. VVA symptoms are a component of Genitourinary Syndrome of Menopause (GSM), a new terminology defined by the International Society for the Study of Women’s Sexual Health and the North American Menopause Society in 2014. Symptoms include dryness, burning sensation, and irritation of the vulvovagina, as well as, sexual problems such as insufficient lubrication, and dyspareunia (pain during sexual intercourse). Lower urinary tract symptoms are also part of GSM and include frequency, recurrence of urinary tract infections, urinary urgency, and stress urinary incontinence. Signs of VVA from physical examination are thinning, drying, and the pallor of the vaginal epithelium due to the decreased ratio of superficial cells to parabasal cells. In addition, a reduction of lactic acid production associates with increased susceptibility to bacterial vaginosis infection [1,2,3]. The vaginal microbiota among postmenopausal women is typically shifted from predominantly *Lactobacillus* species to the higher proportions of anaerobic organisms, including *Mobiluncus* species and *Atopobium vaginae*. These changes in the vaginal bacterial community are associated with the severity of the VVA symptoms [4]. The prevalence of VVA was reported to be as high as 45% among postmenopausal women. The most common complaints were vaginal dryness (55–75%) and pain during intercourse (40–44%) [5,6,7]. However, the majority of patients do not mention their VVA symptoms to health care providers. The European Vulvovaginal Epidemiology Survey of 2160 postmenopausal women reported that using questionnaires indicated a prevalence of severe symptoms of vaginal atrophy, and vulvar atrophy of 66% and 30%, respectively. In contrast, the physical examination confirmed VVA symptoms in as high as 90% in the participants [8]. Under-reporting and subsequent under-treatment of VVA result in more severe and progressive symptoms. This is, especially the case in the Eastern countries where the reported prevalence of VVA was very low, varying from 6-80% among studies conducted in Japan, Sri Lanka, Singapore, and India. However, other data from these studies indicate that the VVA prevalence may be much higher. These surveys report a very high prevalence of “avoiding intimacy” (54–77%) and “loss of interest in sex” (71–91%) among postmenopausal women [9]. VVA not only affects on the quality of life, sexual health, and couple relations, but is also associated with increased risk of depression and anxiety among postmenopausal women [10].

The standard treatment for VVA is hormonal therapy, which can be administered as systemic menopausal hormone therapy (MHT), local estrogen therapy, as well as through other promising novel hormonal regimens such as ospemifene and vaginal DHEA [2,3,11,12]. However, both ospemifene and vaginal DHEA are not yet available in Thailand. Moreover, the study of Women Initiative Study in the systemic MHT study revealed a significantly increased risk of coronary heart disease, invasive breast cancer, and stroke among patients that received conjugated equine estrogen combined with medroxyprogesterone acetate [13]. The announcement of the WHI studies in 2002 resulted in the declining use of systemic MHT by more than 50% and caused a reluctant presumption in both physicians and women worldwide [14]. Although, vaginal estrogen is the most effective treatment for VVA, the examination of the effectiveness and endometrial safety of this therapy has been limited with the longest study period of only one year. Moreover, the only vaginal estrogen available in Thailand is the estriol-lactobacilli combination [15,16]. Non-hormonal therapy and alternative treatment of VVA symptoms are often the preferred choice for many patients. These include lifestyle modifications, encouraging sexual activity, use of vaginal lubricants and vaginal moisturizers, vaginal oxytocin gel, vaginal laser therapy, and vitamin D supplementation [17,18,19,20,21,22].

Vitamin D, a lipid-soluble steroid, enhances the absorption of calcium and phosphate thereby promoting a healthy musculoskeletal system. The Endocrine Society’s Practice Guidelines on Vitamin D, published in 2011, describes the range of serum levels of vitamin D seen in patients, Vitamin D deficiency was defined as a serum level of 25-hydroxyvitamin D (25(OH)D) < 20 ng/mL, insufficiency as 21 to 29 ng/mL, and sufficiency level of at least 30 ng/mL [23]. The adequate level of vitamin D attenuates the rising of serum parathyroid hormone (PTH) level, leading to a plateau of bone resorption [24]. Furthermore, vitamin D insufficiency is associated with several health problems including chronic infections, autoimmune disease, and malignancy [25,26,27,28]. Recently, connections have been identified between vitamin D insufficiency and female reproductive dysfunction, including polycystic ovarian syndrome (PCOS), uterine leiomyoma, endometriosis, and poor in vitro fertilization (IVF) outcome [29,30]. Vitamin D insufficiency also contributes to the pelvic floor disorder and a thinning of the vaginal epithelium, which are associated with VVA in postmenopausal women [31]. Vitamin D receptors (VDRs) are involved in regulating the development and differentiation of the stratified epithelium of the vagina, as well as, the maturation of vaginal cells. Lee et al. reported that vitamin D positively regulates cell-to-cell junctions through the VDR, Ras homolog gene family (RhoA), and Ezrin pathway. Ezrin protein, extensively expressed in the vaginal wall, regulates actin-binding proteins responsible for plasma membrane interactions and cell-to-cell junctions. These processes help modulate the strength and flexibility of the vaginal membrane. In contrast, RhoA regulates cell motility, epithelial layer adhesion, cytokinesis, and cell polarity [32]. Therefore, vitamin D inadequacy maybe a linked factor to VVA in postmenopausal women.

The primary source of vitamin D is endogenous production following exposure to sunlight. Ultraviolet B radiation from sun exposure causes 7-dehydrocholesterol under the skin to be converted to pre-vitamin D3, and then vitamin D3 (cholecalciferol). Vitamin D3 is metabolized in the liver to 25-hydroxyvitamin D (25(OH)D), the major circulating form of vitamin D and is used to determine an individual’s vitamin D status. The circulating 25(OH)D is subsequently metabolized in kidneys to the more biologically more active form, 1,25-dihydroxyvitamin D [33]. The lack of adequate sun exposure is the leading cause of vitamin D deficiency and insufficiency due to modern living conditions [34,35,36]. Vitamin D from the diet is another important source and oral supplements are typically given as either vitamin D3 (cholecalciferol) or vitamin D2 (ergocalciferol). Vitamin D3 is derived from animal sources such as oily fish; salmon, mackerel and herring; and cod liver oil. In contrast, vitamin D2 is derived from plants and UV irradiated yeast and mushroom.

Vitamin D inadequacy is a global health problem with approximately 50% of adults worldwide estimated to be vitamin D insufficient. Poor vitamin D status is especially prevalent in low- and middle-income countries [37,38]. Among the Asian population, the prevalence of vitamin D insufficiency was reported to be as high as 75% [39]. Based on a nationwide retrospective cohort study in 2019, a prevalence of vitamin D insufficiency among older women of 43.9% was reported. Major factors contributing to insufficient vitamin D in Thai women are sunscreen usage and sun avoidant behavior. In addition, air pollution in urban areas, such as Bangkok, decreases the amount of UVB available for cutaneous vitamin D synthesis. Furthermore, in Thailand, dairy products are not fortified with vitamin D, and very few vitamin D-rich foods are part of the typical Thai diet [40]. Thus, dietary intake of vitamin D in the Thai population is generally low. The Endocrine Society Practice Guideline recommends vitamin D supplementation of 1500–2000 IU per day to maintaining vitamin D adequacy, as judged by a serum level of 25(OH)D ≥ 30 ng/mL, in all adults aged more than 50 years. In the case of known vitamin D deficiency, supplementation of vitamin D at 50,000 IU per week is recommended to provide all the potential skeletal and non-skeletal health benefits [23]. Vitamin D2 (ergocalciferol) at a dose of 20,000 IU per capsule is available in Thailand from the Government Pharmaceutical Organization. However, the effects of Vitamin D2 on VVA in postmenopausal women have not been examined. This study aimed to evaluate the effect of oral vitamin D2 supplementation on VVA in postmenopausal women by measuring the vaginal maturation index (VMI), cytological changes in vaginal smear such as a shift from superficial squamous cells towards intermediate epithelial cells, vaginal pH, and the visual analog scale (VAS) of VVA symptoms. This study was initiated based on a hypothesis that adequate oral vitamin D supplementation could improve the vaginal health in Thai menopausal women which have a high prevalence of vitamin D insufficiency.

## 2. Materials and Methods

A double-blinded, randomized, placebo-controlled trial was conducted to study the effects of oral ergocalciferol on vaginal health including, VMI, vaginal pH, and VAS of VVA symptoms at six and 12 weeks of supplementation. The primary outcome was VMI in the 12 weeks of study compared to vitamin D and the placebo group. The secondary outcomes were vaginal pH, VAS of VVA symptoms, and adverse drug reaction. The study protocol conformed to the principles of the Declaration of Helsinki was approved by the Ethical Clearance Committee on Human Rights Related to Research Involving Human Subjects Faculty of Medicine Ramathibodi Hospital, Mahidol University (MURA2018/90), clinical trial registration number: TCTR20180419001.

### 2.1. Participants

Postmenopausal women presenting VVA symptoms, who visited the outpatient department of Department of Obstetrics and Gynaecology, Faculty of Medicine, Ramathibodi Hospital, Bangkok, a tertiary care and training hospital between February 2018 and May 2019, were enrolled. Informed consent was provided by all participants. Inclusion criteria were menopausal women, absence of menstruation for at least one year, a previous bilateral oophorectomy or serum FSH level more than 40 IU/L. The exclusion criteria were a history of hormonal treatments or vitamin D supplementation within the previous 12 weeks, abnormal PAP smear, active sexually transmitted disease, active urinary tract infection, abnormal uterine bleeding, or the presence of serious medical conditions, including cardiovascular disease, liver failure, and renal failure. In addition, patients with a history of vitamin D allergy or unwillingness to participate in the study protocol were excluded. The sample size was calculated using the randomized controlled trial for continuous data formula [41], based on previous studies of the results of Rad P. et al. [31]. The ratio of treatment and control groups was 1:1 with α-error and β-error of 0.01 and 0.1, respectively. The calculated sample-size was 33 and the final sample-size was 40 for each group, after add-ons of 20% incomplete or missing data.

### 2.2. Randomization, Blinding, and Intervention Protocol

All participants, mostly locals who live in the Bangkok Metropolitan area, were randomly distributed into groups using a computerized permutated block with four randomizations. The participants and investigators were blinded to the group allocation. Participants in the study group received oral vitamin D2 (ergocalciferol) at 20,000 IU with two capsules per week for 12 weeks. The placebo group received two identical placebo capsules per week for 12 weeks. Participants in both groups were encouraged to avoid any other drugs and supplements, such as hormonal therapy, herbal remedies, as well as, vaginal lubricants and moisturizers during the study period. Participants were instructed to lead a normal life, without controlling other confounding factors that have an effect on the serum vitamin D level, including sun exposure, sunscreen application, and their diet.

### 2.3. Data Collection and Measurements

Baseline characteristics were collected at the time of enrollment, including age, menopausal age, body mass index (BMI), parity, current medication, smoking history, drinking history, exercise habits, sexual activity, and VAS of VVA symptoms. In addition, a medical history was taken including hypertension, diabetes mellitus, and dyslipidemia. Participants were categorized as sexually active, having sexual intercourse two times or more per month, and those who exercised regularly, aerobic exercises more than 75 min per week. Pelvic examinations were performed to exclude any genital lesion or infection, the VMI and vaginal pH were subsequently collected. The testing for the serum 25(OH) D level was also performed.

The VMI evaluation was obtained from the bilateral upper one-third of the vaginal wall using Ayre’s spatula. Samples were smeared onto a glass slide and immediately fixed in 95% alcohol. VMIs were examined by an experienced laboratory technician. The percentage of superficial (S), intermediate (I), and parabasal (P) cells were used to calculate the VMI as (1 × S) + (0.5 × I) + (0 × P). The vaginal pH was obtained from the posterior fornix and was measured with a SIEMENS Multistix^®^ (Siemens Healthcare GmbH, Erlangen, Germany). VAS of VVA symptoms were recorded during an interview about dryness, pain, itching, and dyspareunia symptoms following a 10 points scale (0 = absent, 5 = moderate, 10 = severe). All parameters were collected at baseline, six, and 12 weeks. Serum vitamin D was collected and measured using the chemiluminescent immunoassay (CLIA) technology with a LIAISON^®^ (DiaSorin, Saluggia, Italy) at baseline and 12 weeks.

At the six and 12 weeks of the study, all participants visited their physician and the allocated supplement bottle was returned to the investigator to count the remaining capsules. Good compliance was defined as those who were taking more than 80% of the medicine. All adverse effects were recorded during interviewing for any abnormal symptoms.

### 2.4. Statistical Analysis

All statistical analysis was performed using STATA version 15.0 (StataCorp LLC, College Station, TX, USA). The intention-to-treat analysis was used as a method for analyzing the results in this prospective randomized controlled trial study. The baseline characteristics were analyzed and the quantitative variables were tested using the Shapiro-Wilk test for their distribution of data.

The data with normal distribution were presented as mean ± standard deviation (SD). The data with non-normal distribution data were presented as the median, (ranges). The Student *t*-test was used to compare the continuous variables in parametric data. The Mann-Whitney U test was used for comparison of continuous variables in non parametric data. For comparison of the VMI, vaginal pH, and VAS of VVA between time points within the group, the paired *t*-test was used. The statistically significant level was *p*-value < 0.05.

## 3. Results

**Figure 1:**
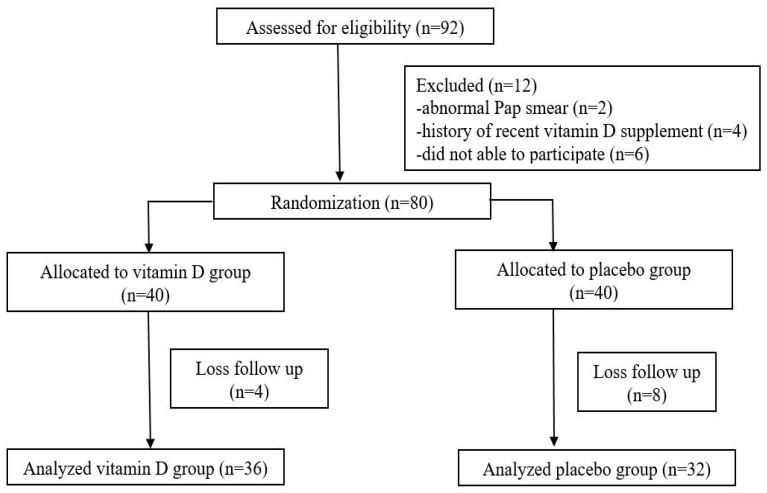
The study flow chart.

Ninety-two postmenopausal women were invited to participate in the study. Twelve women were excluded: Two had an abnormal PAP smear, four had a history of recent vitamin D supplementation, and six were unable to participate in the study. A total of eight participants were included and sixty-eight participants completed the study at the 12th week. There were a total of 12 losses following up at the 12th week, including four in the treatment groups, and eight in the placebo group, as shown in Figure 1.

All of the sixty-eight participants were of good compliance with taking their assigned tablets. The characteristics of participants in both groups showed a not statistically significant difference including VMI, vaginal pH, and VAS of VVA symptoms, as shown in Table 1. After the 12th week of the study, the mean serum 25(OH) D levels were 39.88 ± 13.48 ng/mL and 22.58 ± 7.03 ng/mL in the vitamin D group and placebo group, respectively. Moreover, there was no report of any adverse outcome in both groups.

**Figure 2:**
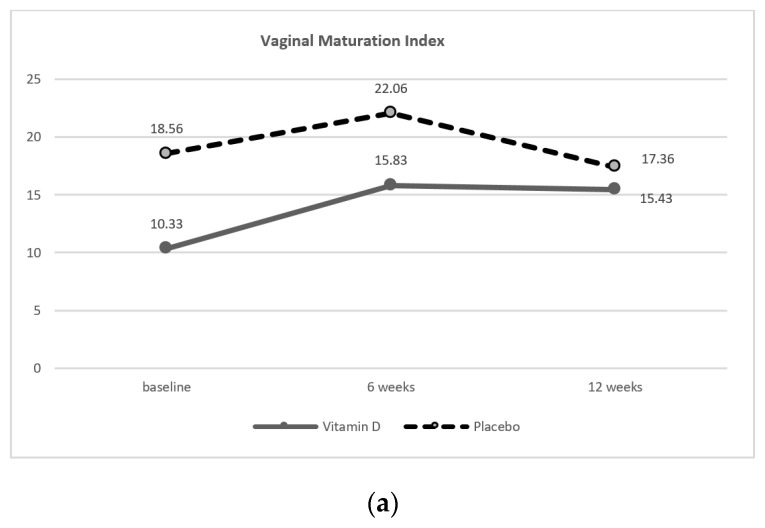

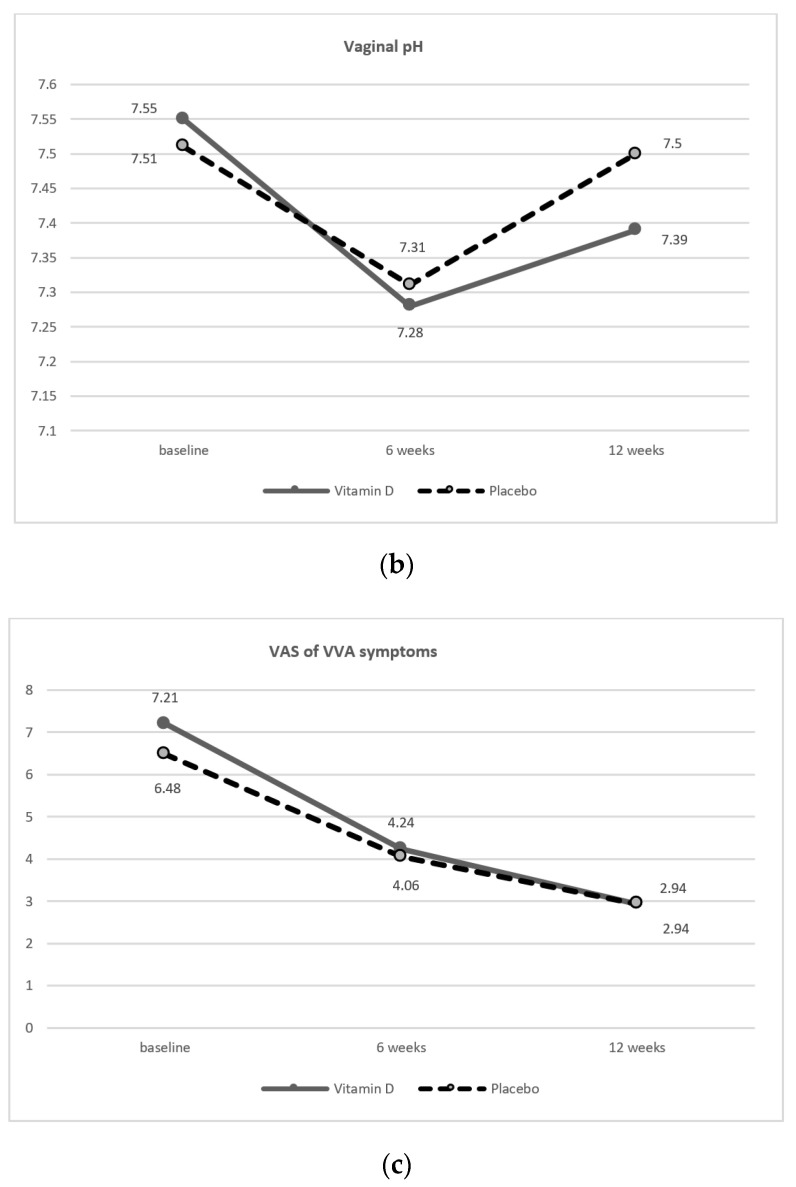
The vaginal health changes between at baseline, six, and 12 weeks with a comparison between the vitamin D group and placebo group: (**a**) Vaginal maturation index (VMI); (**b**) vaginal pH; (**c**) visual analog scale of vulvovaginal atrophy symptoms (VAS of VVA symptoms). VAS—visual analog scale; VVA—vulvovaginal atrophy.

The comparison of vaginal health outcomes, including VMI, vaginal pH, and VAS of VVA symptoms between vitamin D and control groups at baseline, six, and 12 weeks did not reveal a significant difference, see Table 2 and Figure 2.

However, vitamin D caused a significant difference in the mean difference of VMI between baseline and at six weeks (5.5 ± 16.27, *p* < 0.05). Moreover, vitamin D caused a significant improvement in the mean difference of vaginal pH (−0.24 ± 0.38 and −0.21 ± 0.55, respectively) and for VAS of VVA symptoms (−2.97 ± 2.30 and −4.19 ± 2.66, respectively) both at six and 12 weeks of the study, compared to the baseline, as shown in Table 3.

## 4. Discussion

Vitamin D supplementation, in the form of ergocalciferol 40,000 IU per week for 12 weeks in postmenopausal women with VVA symptoms did not affect vaginal health in comparison to placebo therapy. However, ergocalciferol supplements potentially improve most of the vaginal health parameters, including VMI, vaginal pH, and VAS of VVA symptoms, represented as a change in the mean difference compared between baseline, six, and 12 weeks. While such changes were rarely seen in the placebo group except for the VAS of the VVA symptoms, which is a personal-based subjective measure. Moreover, our study was not conducted in women with vitamin deficiency, which may limit the effects of supplementation with ergocalciferol. However, the results were relevant to the previous study by Yildirim B. et al. In their analysis, a cross-sectional study of 60 postmenopausal women, in which 30 participants received an oral 1, 25-dihydroxy vitamin D at 0.5 mcg per day, and another 30 postmenopausal women in a controlled group. Following the participants for one-year revealed that subjects in the vitamin D treatment group had a significant increase in the proportion of superficial cells to basal cells and parabasal cells compared to the placebo group [42]. In addition, a study focused on a vitamin D deficient population by Kaur H. et al. reported the effects of oral cholecalciferol. The effect of cholecalciferol supplementation at 60,000 IU weekly for 10 weeks and then 60,000 IU every three months for six months was examined in 100 menopausal female patients aged 65–78 years with vitamin D deficiency and gynecological diseases, including pelvic floor disorders (PFDs) that effected urinary or fecal incontinence, pelvic organ prolapse (POP), and bacterial vaginosis. After the six-month study, the mean score of the vaginal health index (MVHI) for the pelvic floor patients was significantly improved compared to the placebo and healthy groups [43]. Therefore, the effects of oral vitamin D supplementation on improving vaginal health outcomes may depend on the baseline vitamin D status of the patient, the duration of supplementation, dose, and type of vitamin D supplements such as cholecalciferol 60,000 IU per week for 10 weeks or 1, 25-dihydroxy vitamin D 0.5 mcg per day for a year. From our study, ergocalciferol 40,000 IU per week for 12 weeks can improve the mean difference of vaginal health outcomes, but this effect was not statistically significantly different from the placebo. For the modality of treatment, there are a few reports of vaginal vitamin D administration. Local effects of vitamin D on vaginal health as a report by Rad P. et al. from a double-blind study using the vitamin D vaginal suppository at 1000 IU daily for eight weeks on vaginal atrophy in 40 postmenopausal women. A statistically significant improvement was reported in vaginal health, including VMI, vaginal pH, and dryness symptoms in the treatment group compared to the placebo group [31]. A similar analysis by Keshavarzi Z. et al. reported the beneficial effect of an eight week treatment with vaginal suppositories containing 1000 IU of vitamin D plus 1 mg of vitamin E in improving vaginal atrophy among women with breast cancer receiving tamoxifen [44]. Moreover, the latest narrative systematic review of the effect of vitamin D on vaginal health of menopausal women included six trials with a total of 391 participants, in which two studies were individually randomized controlled trials (RCTs) and four were quasi-randomized. The systematic review indicated that vitamin D alone, in the high doses tested or vaginal use format, appeared to have an effect on vaginal epithelial cells especially superficial cells and promoting a decreased in vaginal pH. The duration of vitamin D use of eight to 10 weeks is sufficient for improvement of vaginal health except for vaginal dryness [45].

One limitation of this analysis is that not all study participants are vitamin D deficient. As a result, changes in vaginal health may not be as apparent comparing the placebo and the vitamin D treatment group. Future research to the optimized dosage, route of treatment, form, and duration of vitamin D treatment has the promise to improve the health effects in postmenopausal women with VVA or genitourinary syndrome of menopause, especially in patients with contraindications for hormonal treatment and vitamin D deficiency.

## 5. Conclusions

Oral vitamin D supplementation, in the form of oral ergocalciferol at 40,000 IU per week for 12 weeks, did not promote a significant difference from the placebo to improve the vaginal health outcomes, including VMI, vaginal pH, and VAS of VVA symptoms in postmenopausal patients with vulvovaginal atrophy. However, vitamin D supplements seemed to improve VMI and vaginal pH both at six and 12 weeks when compared to baseline.

## Figures and Tables

**Table 1:** Baseline characteristics of the participants.

Characteristics	Vitamin D (N = 40)	Placebo (N = 40)
Age (years) ^a^	59.95 ± 5.81	58.33 ± 6.25
Age at menopause ^a^	48.5 ± 5.35	49.5 ± 4.24
Body mass index (kg/m2) ^a^	24.14 ± 3.92	24.63 ± 4.24
Active sexual activity ^b^	19 (51.35%)	18 (48.65%)
Nulliparous ^b^	7 (17.5%)	9 (22.5%)
Having underlying medical diseases	20 (50%)	26 (65%)
Smoking history ^b^	2 (5%)	0 (0%)
Alcohol drinking ^b^	2 (5%)	0 (%)
Regular exercise ^b^	22 (44%)	28 (56%)
Vaginal maturation index ^a^	10.33 ± 19.00	18.56 ± 27.99
Vaginal pH ^a^	7.55 ± 1.02	7.51 ± 0.94
VAS of VVA symptoms ^a^	7.21 ± 2.20	6.48 ± 2.28
Serum 25(OH)vitamin D level (ng/mL) ^a^	24.98 ± 8.25	23.28 ± 7.53

Notes: ^a^ Data expressed as mean ± standard deviation (SD), ^b^ data expressed as a percentage. VAS—visual analog scale; VVA—vulvovaginal atrophy.

**Table 2:** Comparison of the vaginal health measurement between vitamin D group and placebo group at baseline, six, and 12 weeks.

Vaginal Health Measurement	Vitamin D	Placebo	*p*-Value
Vaginal Maturation Index			
Baseline	10.33 ± 19.00	18.56 ± 27.99	0.50
Six weeks	15.83 ± 22.81	22.06 ± 28.54	0.44
12 weeks	15.43 ± 24.87	17.36 ± 26.73	0.81
Vaginal pH			
Baseline	7.55 ± 1.02	7.51 ± 0.94	0.86
Six weeks	7.28 ± 0.94	7.31 ± 0.92	0.89
12 weeks	7.39 ± 0.96	7.50 ± 0.8	0.62
VAS of VVA symptoms			
Baseline	7.21 ± 2.20	6.48 ± 2.28	0.15
Six weeks	4.24 ± 2.44	4.06 ± 2.47	0.76
12 weeks	2.94 ± 2.36	2.94 ± 2.47	0.99

Notes: Data expressed as mean ± standard deviation (SD).

**Table 3:** Comparison of the mean difference of vaginal health measurement between the vitamin D group and placebo group compared between the baseline and at six weeks and between the baseline and at 12 weeks.

Mean Difference	Vitamin D	*p*-Value	Placebo	*p*-Value
Vaginal Maturation Index				
Baseline and at six weeks	5.5 ± 16.27 *	0.04	−3.5 ± 20.42	0.29
Baseline and at 12 weeks	5.1 ± 17.57	0.07	−1.2 ± 16.54	0.65
Vaginal pH				
Baseline and at six weeks	−0.24 ± 0.38 *	<0.05	−0.14 ± 0.54	0.15
Baseline and at 12 weeks	−0.21 ± 0.55 *	0.03	−0.05 ± 0.41	0.52
VAS of VVA symptoms				
Baseline and at six weeks	−2.97 ± 2.30 *	<0.01	−2.47 ± 2.14 *	<0.01
Baseline and at 12 weeks	−4.19 ± 2.66 *	<0.01	−3.59 ± 2.45 *	<0.01

**Notes:** Data expressed as mean ± standard deviation (SD), * *p*-value < 0.05.
